# Exploring the Neuroprotective Mechanism of Curcumin Inhibition of Intestinal Inflammation against Parkinson’s Disease Based on the Gut-Brain Axis

**DOI:** 10.3390/ph16010039

**Published:** 2022-12-27

**Authors:** Lifan Zhong, Benchi Cai, Qitong Wang, Xi Li, Wendi Xu, Tao Chen

**Affiliations:** Department of Neurology, Hainan General Hospital (Hainan Affiliated Hospital of Hainan Medical University), Hainan Medical University, Haikou 570311, China

**Keywords:** curcumin, MPTP, SIRT, NRF2, pyroptosis, Parkinson’s disease, gut-brain axis

## Abstract

Parkinson’s disease (PD) is a chronic progressive neurodegenerative disease commonly seen in aged people, in which gastrointestinal dysfunction is the most common nonmotor symptom and the activation of the gut–brain axis by intestinal inflammation may contribute to the pathogenesis of PD. In a previous study, curcumin was considered neuroprotective in PD, and this neuroprotective mechanism may act by inhibiting intestinal inflammation. Therefore, the aim of this study was to evaluate the effect of curcumin on motor dysfunction and the loss of dopaminergic neurons in a PD mouse model, induced by N-methyl-4-phenyl-1,2,3,6-tetrahydropyridine (MPTP) using open field test and pole test behavioral assessments and the immunofluorescence and Western blot methods. Moreover, the effects of curcumin on gastrointestinal dysfunction, gastric barrier function, pro-inflammatory cytokines, and the SIRT1/NRF2 pathway in intestinal tissues in a PD mouse model were assessed using fecal parameters and intestinal dynamics, immunofluorescence, ELISA, and Western blot. A motor impairment study of an MPTP-induced mouse group prior to treatment with curcumin had a lower total movement distance and a slow average speed, while there was no statistical difference in the curcumin group. After treatment with curcumin, the total movement distance and average speed improved, the tyrosine hydroxylase (TH) rate in the substantia nigra pars compacta (SNpc) and striatum were reduced, the pyroptosis of AIM2 and caspase-1 activations were inhibited, and intestinal inflammatory factors and intestinal inflammation were reduced. Curcumin improved gastrointestinal disorders and gastrointestinal barrier function in the MPTP-induced mice and reversed MPTP-induced motor dysfunction and dopaminergic neuron loss in mice. The above effects may be partly dependent on curcumin activation of the SIRT1/NRF2 pathway in the colon. This study provides a potential opportunity to develop new preventive measures and novel therapeutic approaches that could target the gut–brain axis in the context of PD and provide a new intervention in the treatment of Parkinson’s disease.

## 1. Introduction

Parkinson’s disease (PD) is the second most common neurodegenerative disease after Alzheimer’s disease, and according to statistics there are 7 to 10 million people worldwide with Parkinson’s disease, which poses a serious burden to society [1,2]. The pathology of PD is characterized by the loss of dopaminergic neurons in the dense substantia nigra and abnormal aggregation of α-synuclein [3]. The pathogenesis of PD is still uncertain. The main clinical symptoms of PD are motor and nonmotor impairment. Motor impairments mainly manifest as resting tremor, bradykinesia, myotonia, and postural gait abnormalities. One of the most common nonmotor symptoms is gastrointestinal dysfunction, which includes swallowing difficulties, delayed gastric emptying, and chronic constipation, and the appearance of nonmotor symptoms often precedes motor symptoms by more than 10 years [4,5]. Recent studies suggest that activation of the gut-brain axis by intestinal inflammation may contribute to the pathogenesis of PD [6]. Intestinal inflammation disrupts the physical barrier of the intestine and produces pro-inflammatory factors, such as IL-1β, IL-6, IL-18, and TNFα, which can enter circulation and cross the blood-brain barrier, thereby triggering and exacerbating neuroinflammation [7,8]. The cytokine profiles in other parkinsonisms such as dementia with Lewy bodies (DLB), multiple system atrophy (MSA), and progressive supranuclear palsy (PSP) also play an important role, and inflammatory pathogenesis is also related with other entities’ parkinsonisms [9,10]. Peripheral inflammation may be used as one of the biomarkers of alpha-synucleinopathies [11]. Therefore, in a Parkinson’s disease model, attenuating peripheral inflammation—especially intestinal inflammation—delays neurodegenerative progression and neuroinflammation, suggesting that peripheral inflammation may be a potential target for parkinsonisms [12,13,14,15].

Pyroptosis is an important mechanism of intestinal inflammation [16] in addition to apoptosis, necroptosis, and autophagy. It has been demonstrated that the SIRT1/NRF2 pathway can inhibit pyroptosis in necrotizing colitis [17], acute kidney injury [18], and myocardial ischemia [19]. Sirtuin 1 (SIRT1) is a multifunctional transcriptional regulator involved in the regulation of multiple signaling pathways [20]. It is a multifunction transcriptional regulator involved in the regulation of multiple signaling pathways, including the downstream transcription factor nuclear factor E2-related factor 2 (NRF2), which plays a protective role in inflammatory injury [21] and has neuroprotective effects [22]. It is worth noting that the SIRT1/NRF2 pathway reduces pyroptosis and intestinal inflammation.

Curcumin is a phenolic pigment extracted from the rhizome of turmeric, which is biotransformed to dihydrocurcumin and tetrahydrocurcumin in vivo and is an important active ingredient of turmeric with a wide range of pharmacological effects, such as antitumor, antioxidant, anti-inflammatory, antibacterial, and hypolipidemic properties [23,24,25,26]. In addition, curcumin promotes Parkinson’s disease treatment through multiple pathways, including the prevention of reactive oxygen species (ROS) production, glial cell activation, a-syn aggregation, neuronal cell apoptosis, and iron death [27,28,29,30]. However, poor bioavailability and lack of blood–brain barrier permeability have led to widespread speculation regarding how curcumin might play this role in PD [31]. Can Cui et al. suggested that curcumin may have more important peripheral effects than direct effects on the central nervous system [32]. Curcumin is known to improve intestinal barrier function and reduce inflammation [33,34]. Several studies have shown that curcumin can activate the SIRT1/NRF2 pathway [17,35,36,37,38,39,40]. However, there are no reports of its protective role in Parkinson’s disease. In addition, it is not clear whether curcumin can reduce intestinal inflammation by activating the SIRT1/NRF2 pathway to inhibit pyroptosis and, thus, play an antiparkinsonian role. Therefore, the aim of this study was to investigate the effect of curcumin on motor dysfunction and the loss of dopaminergic neurons as well as the effects of curcumin on gastrointestinal dysfunction, gastric barrier function, pro-inflammatory cytokines, and the SIRT1/NRF2 pathway in intestinal tissues in control, MPTP, MPTP + Cur, MPTP + Cur + Sirtinol, MPTP + Sirtinol and curcumin and sirtinol groups. The information obtained from this study has advanced knowledge and provided useful guidelines on how curcumin can reduce neuroinflammation via the gut–brain axis, and it may be a potential therapeutic approach for Parkinson’s disease.

## 2. Results

### 2.1. Curcumin Improved Motor Impairment in MPTP-Induced Mice

#### 2.1.1. Open Field Test

**Figure 1:**
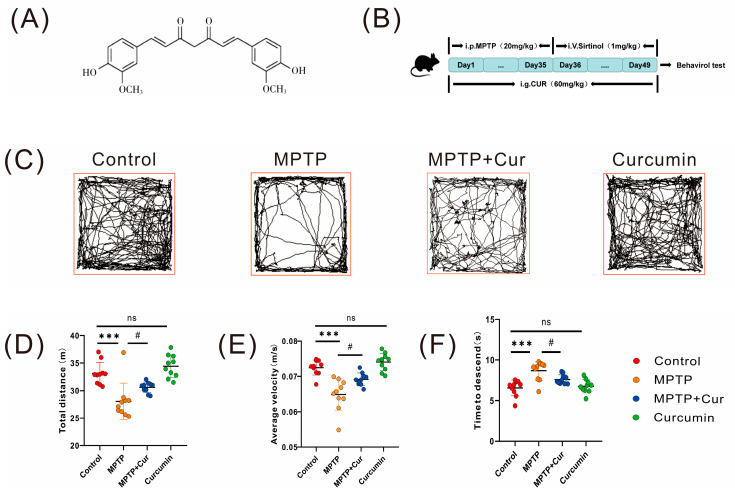
Curcumin treatment ameliorated MPTP-induced dyskinesia in PD mice. Male C57BL/6 mice were injected with curcumin (60 mg/kg) by gavage once daily for 49 days. Mice were injected intraperitoneally with MPTP (20 mg/kg) once every 3.5 days for a total of 10 intraperitoneal injections to establish a PD mouse model. Behavioral tests were performed on day 50 to assess the motor function of mice, and PD pathology in the mice was detected by immunofluorescence and immunoblotting. (**A**) Molecular structure of curcumin; (**B**) experimental design of curcumin intervention on MPTP-induced PD mice; (**C**–**E**) representative traces and quantification of the open field test among control, MPTP, MPTP + Curcumin, and curcumin groups (*n* = 10); (**F**) pole-climbing experiments among the four groups (*n* = 10); All statistical differences were tested using a *t*-test (**D**–**F**). Data are expressed as the mean ± SEM (*n* = 10/group;).The significance is expressed as *** *p* < 0.001 vs. control group. # *p* < 0.05 vs. MPTP group. ns, not significant.

An open field test was performed to assess the amount of exercise and locomotor capacity of the experimental groups. The results showed the movement trajectory patterns of the different experimental groups (Figure 1C). The MPTP-induced mouse group had significantly less total movement distance (*p* < 0.001) and average velocity (*p* < 0.0104) than the control group for the 10 min of activity time, while there were no statistical differences in the curcumin group. Compared with the MPTP group, the mice in the MPTP + Cur group had prolonged exercise (*p* < 0.001) (Figure 1D,E).

#### 2.1.2. Pole Test

Pole tests assess locomotor retardation, and the results showed that the MPTP group significantly prolonged the time required for mice to climb down the pole compared to the control group (*p* < 0.001), indicating that MPTP induced locomotor retardation in mice, while there was no statistical difference in the curcumin group. Mice in the MPTP + Cur group significantly reversed the time required (*p* < 0.0187) for MPTP to induce mice to climb down the pole compared to the MPTP group (Figure 1F).

### 2.2. Curcumin Reduced Dopaminergic Neuronal Degeneration in MPTP-Induced Mice

**Figure 2:**
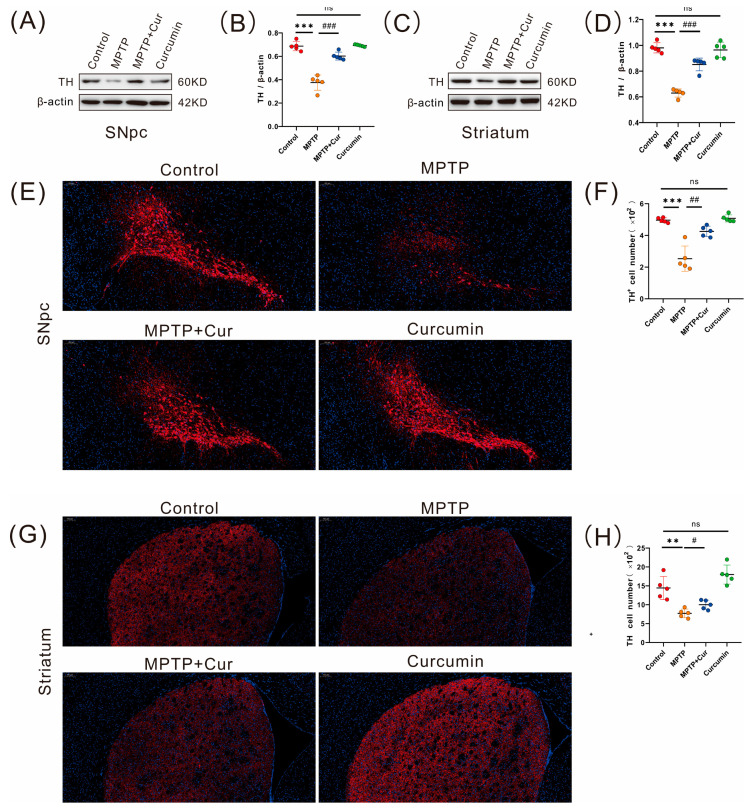
Curcumin treatment reduced MPTP-induced Dopaminergic Neuronal Degeneration in PD mice. (**A**–**D**) Quantification of TH expression in the SN and striatum by Western blot in the four groups (*n* = 5); (**E**,**G**) Representative immunofluorescence images of TH-positive cells in the SN and striatum of the four groups; (**F**,**H**) Quantitative analysis of TH-positive regions in the SN and striatum in the four groups (*n* = 5). The quantitative analysis of the TH-positive regions was performed using ImageJ. All statistical differences were tested using a *t*-test (**B**,**D**,**F**,**H**). Data are expressed as the mean ± SEM (*n* = 5/group).The significance is expressed as ** *p* < 0.01, and *** *p* < 0.001 vs. control group. # *p* < 0.05, ## *p* < 0.01, and ### *p* < 0.001 vs. MPTP group. ns, not significant.

Tyrosine hydroxylase (TH) is the rate-limiting enzyme for dopamine (DA) synthesis [41], and a reduction in TH is one of the key pathological changes in the degenerative necrosis of dopaminergic neurons in Parkinson’s disease. To assess whether curcumin could improve MPTP-induced PD mice, we used Western blot and immunofluorescence analysis to detect TH expression in the SN and striatum. The results showed that TH-positive cells in the SN (*p* < 0.001 and *p* < 0.0021, respectively) and striatum (*p* < 0.001 and *p* < 0.0017, respectively) were significantly reduced in the MPTP group compared with the control group, and there was no statistical difference in the curcumin group. Compared with the MPTP group, the MPTP + Cur group significantly improved the expression of TH in the SN (*p* < 0.001 and *p* < 0.001, respectively) and striatum (*p* < 0.001 and *p* < 0.0161, respectively), and the above experiments confirmed that curcumin could improve the motor symptoms of Parkinson’s disease (Figure 2).

### 2.3. Curcumin Improved Gastrointestinal Dysfunction in MPTP-Induced Mice

**Figure 3:**
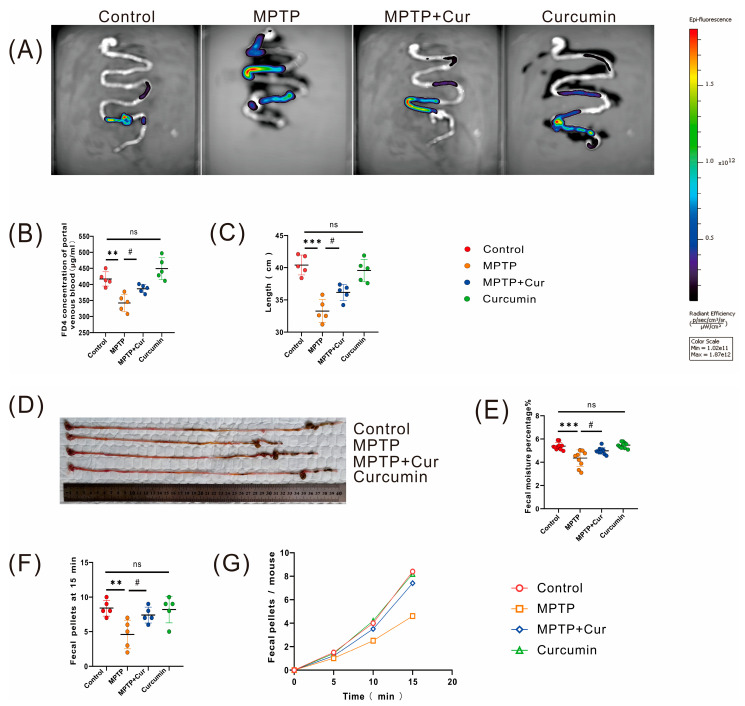
Curcumin treatment ameliorated MPTP-induced gastrointestinal dysfunction in PD mice. (**A**,**B**) Intestinal motility test (*n* = 5/group): (**A**) representative image; (**B**) concentration of isothiocyanate–dextran in portal blood; (**C**,**D**) full-field anatomical images of the gastrointestinal tract of mice (*n* = 5/group); (**E**) fecal water content (*n* = 10 groups); (**F**,**G**) number of 15 min fecal pellets in mice; All statistical differences were tested using the *t*-test (**B**,**C**,**E**,**F**). Data are expressed as the mean ± SEM. Representative results are for one of the independent experiments. ** *p* < 0.01, and *** *p* < 0.001 vs. control group. # *p* < 0.05 vs. MPTP group. ns, not significant.

The most common nonmotor dysfunction in PD is gastrointestinal dysfunction [42]. To assess whether curcumin could improve MPTP-induced gastrointestinal dysfunction in PD mice, we used a gastrointestinal dynamics assay. The results showed representative images of different groups, as shown in Figure 3A. Compared with the control group, gastrointestinal function was impaired in the MPTP group mice (*p* < 0.0014) and unaltered in the curcumin group mice. Intestinal motility was enhanced in the MPTP + Cur group compared to the MPTP group (*p* < 0.0104). In addition, as the amount of intestinal leakage increased, detectable serum FD4 also increased (Figure 3B). The results from the gastrointestinal anatomical map suggest that the mice in the MPTP group had a shortened intestine and an atrophied colon compared to the control group (*p* < 0.001), while the curcumin group had no change in intestinal length and no atrophy of the colon was seen. Compared with the MPTP group, the mice in the MPTP + Cur group showed a slight improvement in intestinal shortening and a decrease in colonic atrophy (*p* < 0.0182) (Figure 3C,D).

Fecal parameters were measured to assess overall gastrointestinal function after MPTP induction. The results showed that the ratio of fecal wet weight to dry weight significantly decreased, and the number of 15 min fecal pellets decreased in the MPTP group, compared with the control group (*p* < 0.001 and *p* < 0.0071, respectively); the ratio of fecal wet weight to dry weight and the number of fecal pellets did not change significantly in the curcumin group. Compared with the MPTP group, the fecal water content of the mice in the MPTP + Cur group increased, and the number of fecal pellets at 15 min increased slightly (*p* < 0.0178 and *p* < 0.0294, respectively) (Figure 3E–G).

**Figure 4:**
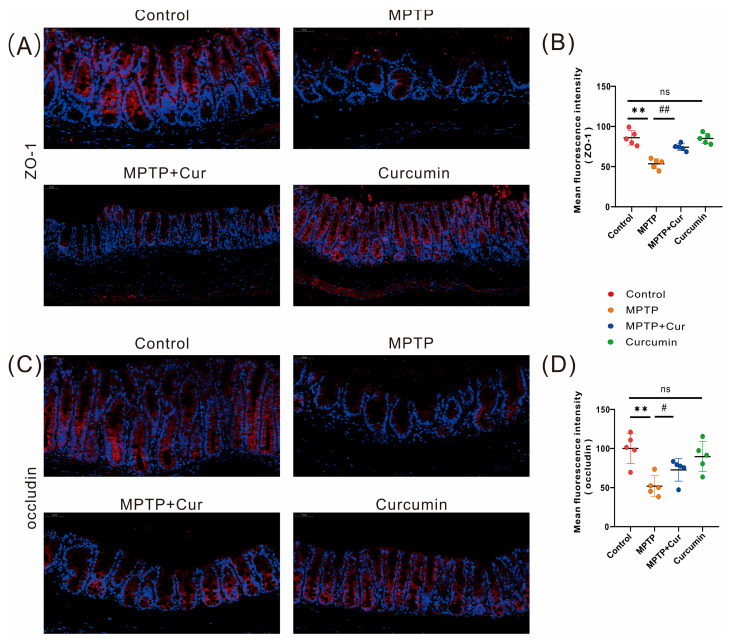
Curcumin treatment ameliorated MPTP-induced gastrointestinal barrier disruption in PD mice. (**A**,**C**) representative immunofluorescence images of ZO-1- and occludin-positive cells in the cecum. (**B**,**D**) Analysis of ZO-1 and occludin fluorescence intensity in the cecum (*n* = 5);The quantitative analysis of ZO-1- and occludin-positive regions was performed using ImageJ. All statistical differences were tested using the *t*-test. Data are expressed as the mean ± SEM (*n* = 5/group). Representative results are for one of the independent experiments. ** *p* < 0.01 vs. control group. # *p* < 0.05 and ## *p* < 0.01 vs. MPTP group. ns, not significant.

To assess whether curcumin ameliorates MPTP-induced gastrointestinal barrier disruption in mice, we examined the immunofluorescence staining of two tight junction proteins, ZO-1 and occludin, and the results showed a reduction in fluorescence intensity and a small amount of protein distribution in the MPTP group compared with the control group, suggesting that the physical barrier of the colon was disrupted (*p* < 0.01 and *p* < 0.01), while there was no significant change in the fluorescence intensity and distribution of the two proteins in the curcumin group when compared with the control group. The intestinal barrier was slightly restored in the MPTP + Cur group (*p* < 0.01 and *p* < 0.046, respectively) (Figure 4A–D).

### 2.4. Curcumin Inhibits Pyroptosis-Mediated Gastrointestinal Inflammation

**Figure 5:**
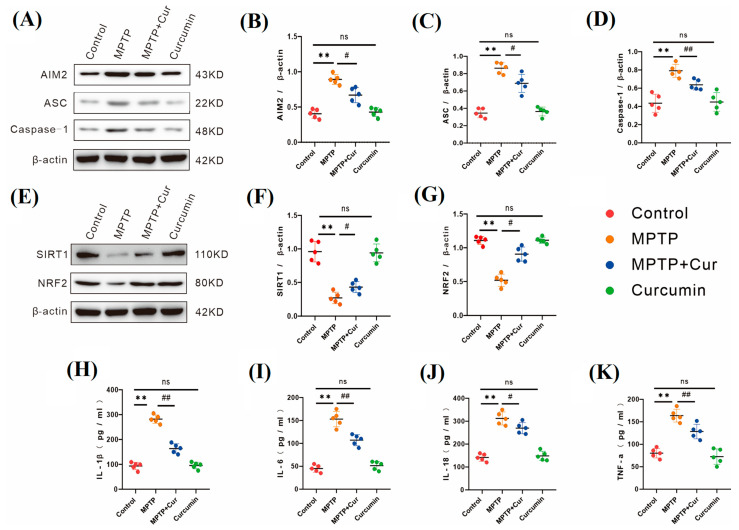
Curcumin ameliorated the inhibition of pyroptosis to attenuate intestinal inflammation and activation of the SIRT1/NRF2 pathway in MPTP-induced model mice. (**A**) Western blot was used to detect AIM2, ASC, and caspase-1 proteins in the four groups of cecum tissues (*n* = 5); (**B**–**D**) analysis of AIM2, ASC, and caspase-1 protein expression in cecum tissues (*n* = 5); (**E**) detection of SIRT1 and NRF2 proteins in the four groups of cecum tissue by Western blot (*n* = 5); (**F**,**G**) analysis of SIRT1 and NRF2 protein expression in cecum tissue (*n* = 5); AIM2, ASC, caspase-1, SIRT1, and NRF2 protein expressions were quantified using ImageJ; (**H**–**K**) the expression levels of pro-inflammatory factors IL-1β, IL-6, IL-18, and TNF-α in intestinal tissue homogenates were analyzed using ELISA (*n* = 5). All statistical differences were tested using the *t*-test (**B**–**D**,**F**–**K**). Data are expressed as the mean ± SEM (*n* = 5/group), with representative results for one of the independent experiments. ** *p* < 0.01 vs. control group. # *p* < 0.05 and ## *p* < 0.01 vs. MPTP group. ns, not significant.

Several studies have suggested that gastrointestinal dysfunction in mice may be due to the inflammation of the gastrointestinal tract caused by cellular pyroptosis [43,44,45,46]. We used protein immunoblotting and an enzyme-linked immunosorbent assay for the detection of pyroptosis-mediated gastrointestinal inflammations. The results are shown in Figure 5A–D. Compared with the control group, the key indicators of pyroptosis (i.e., AIM2, ASC, and caspase-1) and the inflammatory indicators (i.e., IL-1β, IL-6, IL-18, and TNF-α) were elevated in the intestinal tissue of the MPTP group (Figure 5H–K), while the indicators of pyroptosis and the inflammatory indicators did not significantly change in the intestinal tissue of the curcumin group (*p* < 0.01, *p* < 0.01, *p* < 0.01, *p* < 0.01, *p* < 0.01, *p* < 0.01, and *p* < 0.01). Compared with the MPTP group, the pyroptosis death indexes and inflammation indexes were reduced in the MPTP + Cur group, which was statistically different (*p* < 0.05, *p* < 0.0132, *p* < 0.0052, *p* < 0.001, *p* < 0.0011, *p* < 0.0367, and *p* < 0.0062, respectively).

### 2.5. Curcumin Activated the SIRT1/NRF2 Pathway

In a previous study, it was confirmed that the SIRT1/NRF2 pathway plays an anti-inflammatory protective role in a large number of diseases [18,47,48,49,50,51,52,53,54,55]. Antipyroptosis is one of the roles of this pathway. Curcumin ameliorated pyroptosis and activated the SIRT1/NRF2 pathway, which we detected using protein immunoblotting. The results were as follows (Figure 5E–G): the expression of SIRT1 and NRF2 in intestinal tissues was significantly reduced in the MPTP group compared to the control group (*p* < 0.01 and *p* < 0.016, respectively), and no significant changes were observed in the curcumin group. The expression of SIRT1 and NRF2 in intestinal tissues was slightly increased in the MPTP + Cur group compared with the MPTP group, which was statistically different (*p* < 0.01 and *p* < 0.05, respectively).

### 2.6. Curcumin Reduced Intestinal Inflammation by Activating the SIRT1/NRF2 Pathway to Inhibit Pyroptosis

**Figure 6:**
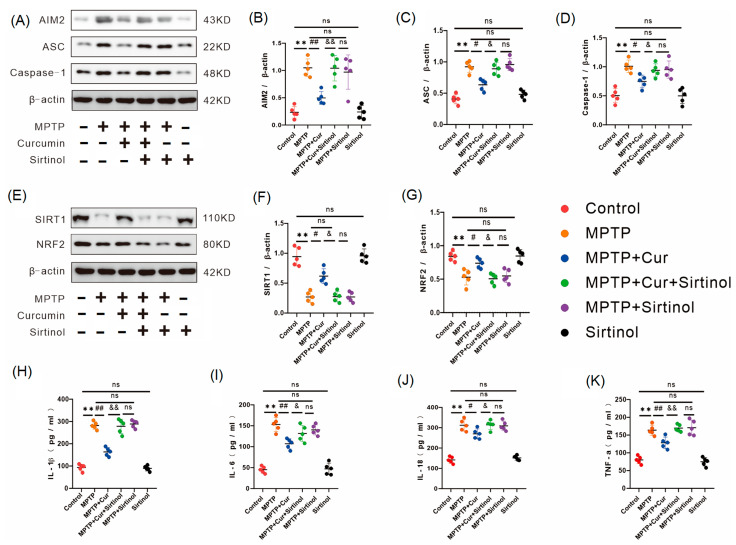
Curcumin inhibited cellular scorching by activating the SIRT1/NRF2 pathway, thereby reducing intestinal inflammation. (**A**) Western blot was used to detect AIM2, ASC, and caspase-1 proteins in the six groups of cecum tissue (*n* = 5); (**B**–**D**) analysis of AIM2, ASC, and caspase-1 protein expression in cecum tissue (*n* = 5); (**E**) detection of SIRT1 and NRF2 proteins in the six groups of cecum tissue using Western blot (*n* = 5); (**F**,**G**) analysis of SIRT1 and NRF2 protein expression in the six groups of cecum tissue (*n* = 5); AIM2, ASC, caspase−1, SIRT1, and NRF2 protein expressions were quantified using ImageJ; (**H**–**K**) the expression levels of pro-inflammatory factors IL-1β, IL-6, IL-18, and TNF-α in intestinal tissue homogenates were analyzed using ELISA (*n* = 5). All statistical differences were tested using the *t*-test (**B**–**D**,**F**–**K**). Data are expressed as the mean ± SEM (*n* = 5/group), with representative results for one of the independent experiments. ** *p* < 0.01 vs. control group. # *p* < 0.05 and ## *p* < 0.01 vs. MPTP group. & *p* < 0.05 and && *p* < 0.01 vs. MPTP + Cur group. ns, not significant.

Based on these results, we further used sirtinol and observed whether the anti-inflammatory effect of curcumin could be reversed. We used protein immunoblotting to detect SIRT1 and NRF2 protein indicators (Figure 6E). The results were as follows: SIRT1 and NRF2 protein expression was reduced in the intestinal tissues of the MPTP + Cur + Sirtinol (SIRT1 inhibitor) group compared to the MPTP + Cur group. There was no significant change in the sirtinol group compared to the control group (*p* < 0.001 and *p* < 0.0014, respectively) (Figure 6F,G).

We then used protein immunoblotting and an enzyme-linked immunosorbent assay to detect the following results: the key indicators of pyroptosis (i.e., AIM2, ASC, and caspase-1) were elevated in the intestinal tissues of the MPTP + Cur + Sirtinol group compared with the MPTP + Cur group (Figure 6A–D), and the inflammatory indicators (i.e., IL-1β, IL-6, IL-18, and TNFα) were all elevated. No significant changes were observed in the sirtinol group compared with the control group (*p* < 0.0016, *p* < 0.05, *p* < 0.0263, *p* < 0.01, *p* < 0.0421, *p* < 0.0174, and *p* < 0.0013, respectively) (Figure 6H–K).

### 2.7. Curcumin Ameliorated Gastrointestinal Dysfunction by Reducing Intestinal Inflammation in MPTP-Induced Mice

**Figure 7:**
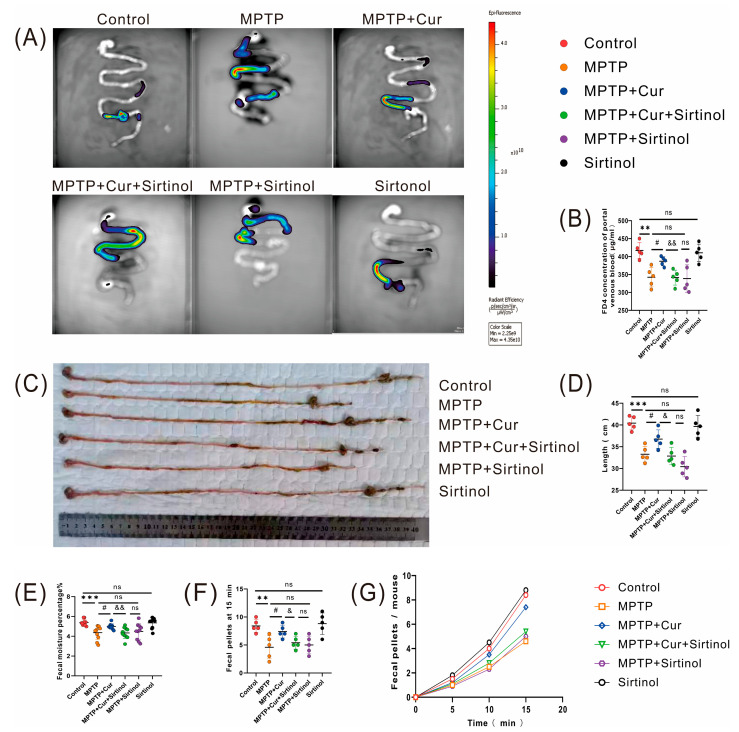
Curcumin ameliorated gastrointestinal dysfunction by reducing intestinal inflammation in MPTP-induced mice. (**A**,**B**) Intestinal motility test (*n* = 5/group): (**A**) representative image, (**B**) concentration of isothiocyanate–dextran in portal venous blood; (**C**,**D**) full-field anatomical images of mouse gastrointestinal tract (*n* = 5/group); (**E**) fecal water content (*n* = 10 groups); (**F**,**G**) number of 15 min fecal pellets in mice;All statistical differences were tested using the *t*-test using (**B**,**D**–**F**). Data are expressed as the mean ± SEM (*n* = 5/group). Representative results are for one of the independent experiments. ** *p* < 0.01 and *** *p* < 0.001 vs. control group. # *p* < 0.05 vs. MPTP group. & *p* < 0.05 and && *p* < 0.01 vs. MPTP + Cur group. ns, not significant.

We used an intestinal dynamics assay to investigate gastrointestinal dysfunction, and the results were as follows: the MPTP + Cur + Sirtinol group had slower significant intestinal peristalsis and less permeability than the MPTP + Cur group (*p* <0.01). There was no significant difference in the sirtinol group compared to the control group (Figure 7A,B). The results of the gastrointestinal autopsy were as follows: compared with the MPTP + Cur group, the mice in the MPTP + Cur + Sirtinol group showed increased intestinal length and no atrophy of the colon (*p* < 0.0166), and the results of the sirtinol group after treatment were not significantly different from those of the MPTP group (Figure 7C,D).

The results of the fecal parameters showed a significant decrease in fecal water content and fecal particle number in the MPTP + Cur + Sirtinol group compared to the MPTP + Cur group (*p* < 0.01 and *p* < 0.0242, respectively). There was no significant difference in the sirtinol group compared to the control group (Figure 7E–G).

**Figure 8:**
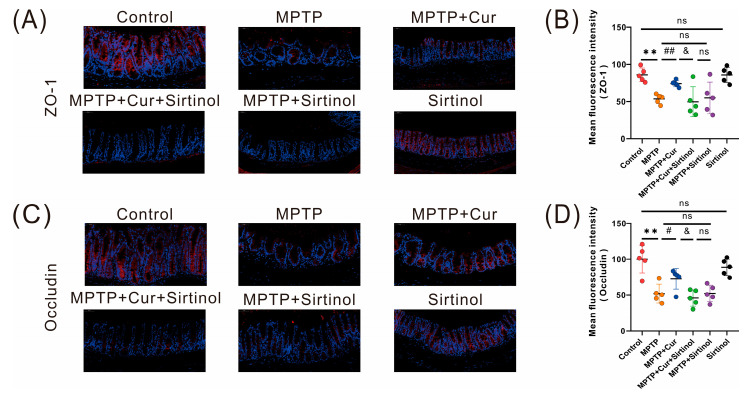
Curcumin ameliorated gastrointestinal barrier disruption by reducing intestinal inflammation in MPTP-induced mice. (**A**,**C**) representative immunofluorescence images of ZO-1- and occludin-positive cells in the cecum (*n* = 5); (**B**,**D**) analysis of ZO-1 and occludin fluorescence intensity in the cecum. The quantitative analysis of ZO-1- and occludin-positive regions was performed using ImageJ. All statistical differences were tested using the *t*-test using (**B**,**D**). Data are expressed as the mean ± SEM (*n* = 5/group). Representative results are for one of the independent experiments. ** *p* < 0.01 vs. control group. # *p* < 0.05 and ## *p* < 0.01 vs. MPTP group. & *p* < 0.05 vs. MPTP + Cur group. ns, not significant.

The immunofluorescence analysis detected the gastrointestinal barrier proteins ZO-1 and occludin; the results showed that the distribution and fluorescence intensity of ZO-1 and occludin proteins significantly decreased in the MPTP + Cur + Sirtinol group compared with the MPTP + Cur group (*p* < 0.0290 and *p* < 0.0113, respectively), and there was no significant difference in the sirtinol group compared with the control group (Figure 8A–D). In conclusion, the gastrointestinal function of the MPTP + Cur mice could not be improved after sirtinol treatment, further confirming that curcumin could reduce intestinal inflammation and, thus, improve gastrointestinal dysfunction in Parkinson’s disease mice by activating the SIRT1/NRF2 pathway.

### 2.8. Neuroprotective Effects of Curcumin Were Dependent on the Gut–Brain Axis

**Figure 9:**
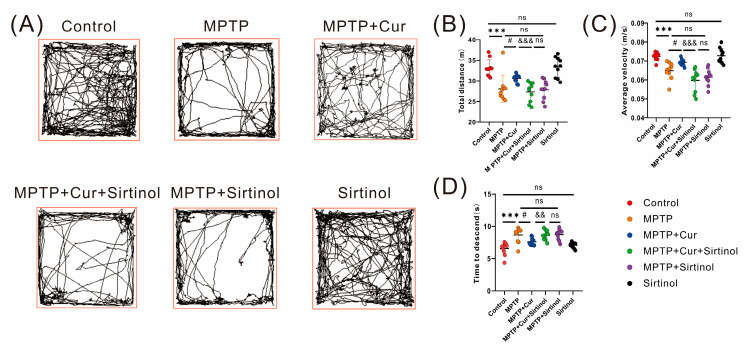
The neuroprotective effect of curcumin was dependent on the gut–brain axis. (**A**–**C**) Representative traces and quantification of the open field test among the control, MPTP, MPTP + Cur, MPTP + Cur + Sirtinol, MPTP + Sirtinol, and sirtinol groups (*n* = 10); (**D**) quantification of the performance in the pole test among the six groups (*n* = 10); All statistical differences were tested using the *t*-test (**B**–**D**). Data are expressed as the mean ± SEM (*n* = 10/group). Representative results are for one of the independent experiments. *** *p* < 0.001 vs. control group. # *p* < 0.05 vs. MPTP group. && *p* < 0.01 and &&& *p* < 0.001vs. MPTP + Cur group. ns, not significant.

The results of the open field test are shown in Figure 9A, and the results revealed that the MPTP + Cur + Sirtinol group resulted in a significant reduction in the total distance traveled and time spent active during 10 min of exercise compared to the MPTP + Cur group (*p* < 0.001 and *p* < 0.001) (Figure 9B,C). There was no statistical difference in the sirtinol group compared to the control group. The results of the pole test showed (Figure 9D) that the treatment in the MPTP + Cur + Sirtinol group led to a prolonged time required for the mice to climb down to the bottom of the pole compared to the MPTP + Cur group. There was no statistical difference in the sirtinol group compared to the control group (*p* < 0.05).

**Figure 10:**
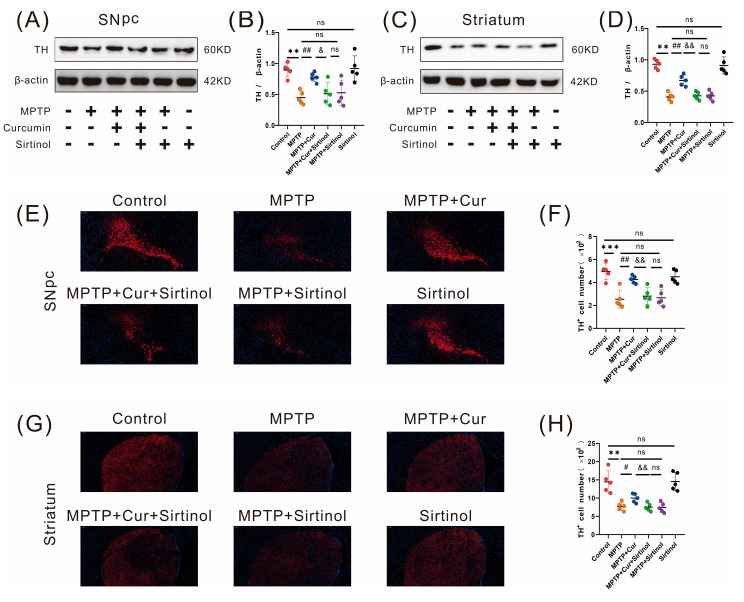
The neuroprotective effect of curcumin was dependent on the gut–brain axis. (**A**−**D**) Quantification of the TH expression in the SN and striatum using Western blot in the six groups; (**E**,**G**) representative immunofluorescence images of TH—positive cells in the SN and striatum of the six groups; (**F**,**H**) Quantitative analysis of TH—positive regions in the SN and striatum in the six groups (*n* = 5). The quantitative analysis of the TH—positive regions was performed using ImageJ. All statistical differences were tested using the *t*-test (**B**,**D**,**F**,**H**). Data are expressed as the mean ± SEM ( *n* = 5/group). Representative results are for one of the independent experiments. ** *p* < 0.01 and *** *p* < 0.001 vs. control group. # *p* < 0.05 and ## *p* < 0.01 vs. MPTP group. & *p* < 0.05, && *p* < 0.01 vs. MPTP + Cur group. ns, not significant.

We used Western blot and immunofluorescence detection to analyze the TH grayscale values and cell counts in the SN and striatum. The results suggested that treatment in the MPTP + Cur + Sirtinol group resulted in a significant decrease in the gray value of TH (*p* < 0.0147 and *p* < 0.001, respectively) and cell number of TH (*p* < 0.001 and *p* < 0.001) compared to the MPTP + Cur group. There was no statistical difference in the sirtinol group compared to the control group (Figure 10A–H).

In conclusion, if curcumin fails to activate the SIRT1/NRF2 pathway to attenuate intestinal inflammation, dopaminergic neuronal degeneration and motor dysfunction in the MPTP-induced mouse model cannot be ameliorated. It is suggested that curcumin ameliorates motor impairment in Parkinson’s disease mice dependent on the gut–brain axis.

## 3. Discussion

There is a large body of research suggesting a two-way communication between the gut and the brain [56,57]. This role is often referred to as the gut–brain axis [58]. The gut–brain axis network consists of multiple connections, including the vagus nerve, bacterial metabolites, the immune system, and inflammation. Upon the classical Braak hypothesis that the pathology of PD initiates in the intestinal tract, it has been suggested that α-synuclein spreads from the intestinal nervous system to the brain and dorsal motor nucleus of the vagus in the brainstem via the vagus nerve [59]. Previous studies using vagotomy were used to suggest that α-synuclein may occur from the gut through the vagus nerve [60]. The first report on alterations in the gut microbiota composition in PD was published by Scheperjans et al., who described the associations between the alteration of the composition of gut microbiota and the clinical phenotype of PD [61]. Their study indicates that the fecal abundance of Prevotella in PD patients showed a reduction of 77.6% compared with controls, and patients with a postural instability had a higher abundance of Enterobacteriaceae as compared to tremor-predominant PD patients. Gut microbiome dysbiosis may be one of the factors leading to increased intestinal permeability and intestinal barrier disruption, and this view was confirmed in a model of Parkinson’s disease [62,63,64]. Moreover, a series of studies on PD intestinal tissue suggest alterations in the markers of immune cell populations [65,66]. Compared to other immune cell populations, gut Treg cells have gained wide attention for their ability to maintain self-tolerance and immune homeostasis [67]. In summary, based on the gut–brain axis, exploring potential therapeutic targets for PD is feasible.

Currently, a growing body of evidence supports that curcumin treatment can slow Parkinson’s disease progression. Curcumin is considered a potential PD therapeutic regimen due to its strong antioxidant and anti-inflammatory activities. Previous studies have shown that curcumin was first biotransformed to dihydrocurcumin and tetrahydrocurcumin, which requires the participation of curcumin metabolic microorganisms and curcumin convertase [68,69]. The microbial metabolism of curcumin biotransforms via a two-step reduction, curcumin being converted NADPH-dependently into an intermediate product, dihydrocurcumin, and then the final product, tetrahydrocurcumin [70]. Curcumin convertase belongs to the medium-chain dehydrogenase/reductase superfamily. The previous findings suggest that curcumin-glucuronoside, dihydrocurcumin-glucuronoside, tetrahydrocurcumin-glucuronoside, and tetrahydrocurcumin are major metabolites of curcumin in vivo [71]. Interestingly, there is evidence that curcumin metabolites exhibit similar or superior potency to curcumin and have therapeutic effects on neurodegenerative diseases [72,73]. Notably, curcumin and its metabolites reversed the depletion of dopamine (DA) and DOPAC (3,4-dihydroxy phenyl acetic acid) induced by MPTP in mice [74]. However, more studies have shown that tetrahydrocurcumin, as a free radical agent, has advantages over curcumin and has been shown to have therapeutic effects in neurodegenerative diseases. However, curcumin’s pharmacological effects were hindered by its pharmacokinetic properties, mainly due to the fact of its difficulty being absorbed into the blood and its low bioavailability.

Given the growing reports that the gut–brain axis plays an important role in the pathogenesis of PD, we established a mouse model of PD by intraperitoneal injection of MPTP to assess the protective effect of curcumin treatment on PD. The study showed that curcumin activated the SIRT1/NRF2 pathway and inhibited AIM2 inflammasome-mediated pyroptosis. Concurrently, curcumin could increase the expression of ZO-1 and occluding as well as improve the effect on gut barrier function and gastrointestinal dysfunction. Finally, curcumin reversed dyskinesia and the loss of dopaminergic neurons in an MPTP-induced Parkinson’s disease mouse model.

Neuroinflammation in PD is always accompanied by intestinal inflammation [7,15,75,76], and the inflammatory vesicle-associated pyroptosis mechanism is one of the mechanisms leading to the pathogenesis of intestinal inflammation [16,44,77,78,79,80,81]. Pyroptosis, also known as cellular inflammatory necrosis, is a new type of programmed inflammatory cell death, where the formation of inflammatory vesicles is followed by the secretion and induction of inflammation through the activation of caspase-1 and the pro-inflammatory cytokines interleukin (IL)-1β and IL-18, which are involved in innate immunity to specific pathogens, environmental stimuli, and host cell damage [43]. Several sensors can trigger the formation of inflammatory vesicles, among which AIM2 (missing in melanoma 2) is a cytoplasmic sensor of double-stranded DNA from pathogens or damaged organelles. It recruits ASC (apoptosis-associated spot-like protein containing CARD) and caspase-1 to form AIM2 inflammatory vesicles [82,83,84]. It has been suggested that AIM2 may play a role in neurodegenerative diseases [85,86], ischemic stroke [87], autoimmune diseases [88], cancer, etc. [89]. 

Among others, pyroptosis is associated with neuroinflammatory diseases such as multiple sclerosis, Alzheimer’s disease, and Parkinson’s disease, as well as anxiety and depression-like disorders [44,90]. There is evidence that there is an interaction between neuroinflammation and the activation of inflammatory vesicles [91]. The present study found that inflammatory vesicles and pyroptosis pathways are upregulated in PD intestinal tissue expression. This is consistent with our findings that MPTP induced a significant increase in the expression of AIM2/ASC and caspase-1 in the intestinal tissues of model mice, and that curcumin treatment downregulated these proteins and inhibited pyroptosis. The use of SIRT1 inhibitors may lead to a loss of the protective capacity of curcumin, suggesting that curcumin may ameliorate the intestinal tract in PD through the SIRT1/NRF2 pathway via inflammation and pyroptosis.

There are no relevant drugs or preventive measures involving the gut–brain axis widely used in the clinic, and this study suggests that curcumin may be a potential drug candidate. Curcumin is a diarylheptanoid derivative found in turmeric, which is widely used as a colorant, spice, and food additive. Some studies suggest that curcumin plays a protective role in organs such as the gastrointestinal tract, heart, liver, and nerves, with anti-inflammatory, antioxidant, and immune-enhancing properties [92]. Numerous studies have shown that curcumin may improve Parkinson’s disease [69,93,94,95,96,97,98,99], but the exact mechanism has not been fully clarified, while some studies suggest that curcumin improves intestinal inflammation, possibly by activating the SIRT1/NRF2 pathway. In this study, we used curcumin as a therapeutic regimen to reveal that curcumin activates the SIRT1/NRF2 pathway to inhibit intestinal pyroptosis, reduce intestinal inflammation, and improve intestinal permeability, thus elucidating that the neuroprotective mechanism of curcumin against PD depends on the intestine–brain axis.

It is well known that the gut microbiota mediates the transformation of curcumin, but explorations of this fact are limited in our research. It is worth noting that from the interaction between curcumin and gut microbiota, the regulation of intestinal microflora by curcumin and the biotransformation of curcumin by gut microbiota are both potentially crucial for curcumin activity [100,101]. The next step in our investigation sought to elucidate the interaction between curcumin and gut microbiota and the effect of interaction on biotransformation of curcumin. This will be the subject of our future study.

## 4. Materials and Methods

### 4.1. Animals and Treatments

Male C57BL/6J mice (8 weeks, weight 20 ± 5 g) were provided by Hunan Slaughter Jingda Laboratory Animal Co., Ltd. (SCXK (Xiang) 2019-0004, Changsha, China). The mice were acclimatized and kept in a standard feeding environment (pathogen-free, 24 ± 2 °C, 55 ± 10% humidity, 12–12 h light–dark cycle) for one week before the formal experiments, with free access to food and water. All animal procedures were performed in accordance with the National Institutes of Health Guide for the Care and Use of Laboratory Animals (Guide No. 55 issued by the Ministry of Health, revised edition 1998) and approved by the Ethics Committee of Hainan Hospital, Hainan Medical College (approval number [2022] 203). All the mice were euthanized by 10% chloral hydrate (300 mg/kg) anesthesia followed by cervical dislocation prior to sacrifice. No mice exhibited signs of peritonitis following the administration of 10% chloral hydrate during the study. Disease severity was scored in a semiquantitative fashion described as follows: 0 = no abnormal clinical sign; 1 = ruffled fur but lively; 2 = ruffled fur, moving slowly, and sick; 3 = ruffled fur, squinted eye, hardly moving, down, and very sick; 4 = moribund; and 5 = dead. Humane endpoints (a clinical score of 4 was used as the humane endpoint) were necessary for mice that survived at the conclusion of the experiment.

Mice were randomly divided into seven groups, including the control, curcumin, MPTP, MPTP + Cur, MPTP + Cur + Sirtinol, MPTP + Sirtinol, and sirtinol groups, with 10 mice in each group for 70 mice in total. MPTP (Sigma-Aldrich, St. Louis, MO, USA) was induced to establish a PD mouse model as in [102]. From day 1 to day 35 of the experiment, the model mice were given intraperitoneal injections of MPTP (dissolved in saline) (20 mg/kg) every 3.5 days/injection, for a total of 10 injections. The dose of curcumin was selected based on a previous study [103]. Starting from day 1 of the experiment, curcumin (60 mg/kg) (Macklin, Shanghai, China) was administered by gavage daily for 49 d in the curcumin, Curcumin+MPTP and Curcumin + MPTP + Sirtinol groups, and an equal volume of gum arabic solution was given to the other groups (Macklin, Shanghai, China). Sirtinol (MedMol, Shanghai, China) inhibited SIRT1 to establish pathway inhibition. Sirtinol solution (dissolved in saline) (1 mg/kg) was given in the tail vein for 2 weeks from day 36 to day 49 of the experiment in the MPTP + Curcumin + Sirtinol and sirtinol groups, and an equal volume of saline via tail vein was given in the other groups.

### 4.2. Open Field Test

The mice were placed in a white square field (45 cm × 45 cm × 50 cm), and their behavior in the open field for 10 min was recorded continuously with a video camera (Sony, Tokyo, Japan) located above the field. The results of the open field test were analyzed using a SMART 3.0 small animal behavior video recording and analysis system (Smart 3.0 system). We monitored the trajectory, total distance, and average speed of the mice during the 10 min open field test. The open field site was cleaned with 70% alcohol before use and dried between tests.

### 4.3. Pole Test

The pole test was conducted according to a relevant behavioral protocol. Mice were acclimated to their environment 3 days before the test and the experiment was performed on day 1 after treatment. During the test, head-up mice were placed on top of a rough-surfaced pole (1 cm in diameter and 50 cm in height) and then climbed down the pole. The time it took for the mice to climb down the pole was recorded. If the mice stopped midway or climbed up in reverse, they were required to retest. All mice were formally tested after 5 days of training, and the average time was tested to analyze the motor function of the mice.

### 4.4. Fecal Parameter Measurement

Each mouse was placed individually in a clean cage wiped with 75% alcohol in advance. The timing was started, and the number of fecal pellets collected at 0, 5, 10, and 15 min was recorded [41]. The feces were collected immediately after excretion and weighed to obtain the fecal excretion, i.e., wet weight; after drying at 65 °C for 12 h, the feces were weighed again to obtain the dry weight, and, finally, the fecal water content was calculated. Water content of feces = dry weight of feces/wet weight of feces × 100.

### 4.5. Intestinal Motility Test

To assess intestinal mobility and permeability, we used fluorescein isothiocyanate–dextrose based on relevant experiments [104,105]. The mice were given 200 mg/kg FD4 (Sigma-Aldrich, St. Louis, MO, USA) solution by gavage after 24 h of food fasting intervention. Three hours later, the mice were anesthetized with 10% chloral hydrate intraperitoneally, and blood was taken from the portal vein and immediately centrifuged at 3000 rpm, 4 °C, for 10 min, and the supernatant was taken. The blood FD4 concentration was then quantified using a fluorescence spectrophotometer with an excitation wavelength of 488 nm and an emission wavelength set at 520 nm. The FD4 concentration was calculated from the root standard curve in µg/mL.

### 4.6. Immunofluorescence 

The mice were executed after the behavioral tests and perfused with 4% paraformaldehyde. The brain and cecum were extracted, dehydrated in 30% sucrose solution, OCT-embedded, and serially sectioned at a 25 µm thickness. The sections were blocked, incubated with primary antibodies, and then treated with fluorescent or horseradish peroxidase (HPR)-labeled secondary antibodies. The following antibodies were used: anti-TH antibody (Servicebio, Wuhan, China), anti-ZO-1 antibody (Servicebio, Wuhan, China), anti-occludin antibody (Servicebio, Wuhan, China), and Cy3-conjugated donkey anti-rabbit Ig (Servicebio, Wuhan, China).

### 4.7. Western Blotting

The tissues were homogenized in a precooled RIPA lysis solution, followed by placing the homogenate on ice for 30 min, and then centrifuged at 2000 rpm, 4 °C, for 10 min; the supernatant was then collected, which was the total protein solution. The undenatured protein solution was taken and the protein concentration was measured using a BCA protein concentration assay kit (Beyotime, Shanghai, China) (refer to the kit’s instructions for the specific method). The protein solution was added to 5 * reduced protein loading buffer at a ratio of 4:1 and denatured in a boiling water bath for 15 min. The total protein of each sample was electrophoresed by 10% SDS-PAGE with PVDF (0.22 µm) membrane transfer. The membranes were closed with 5% skim milk powder, incubated with the primary antibody at 4 °C overnight, and then incubated with a secondary antibody of HRP. The protein bands were visualized using a super-enhanced chemiluminescence (ECL) detection reagent (Bio-Rad^®^, Hercules, CA, USA) and gel imaging system (Tanon, Shanghai, China). Band intensity quantification was performed using ImageJ (version 1.8.0, NIH) software. The following antibodies were used: anti-TH antibody (Servicebio, Wuhan, China), anti-SIRT1 antibody (Abcam, Cambridge, MA, USA), anti-NRF2 antibody (Abcam, Cambridge, MA, USA), anti-b-actin antibody (Abcam, Cambridge, MA, USA), anti-AIM2 antibody (Cell Signaling Technology, Berkeley, CA, USA), anti-ASC antibody (Cell Signaling Technology, Berkeley, CA, USA), anti-caspase-1 antibody (Cell Signaling Technology, Berkeley, CA, USA), and goat anti-rabbit antibody (Cell Signaling Technology, Berkeley, CA, USA).

### 4.8. Enzyme-Linked Immunosorbent Assay

The expression of the inflammatory markers IL-1b, IL-6, IL-18, and TNF-a in the intestinal tissues (homogenates) of each group of mice was detected by enzyme-linked immunosorbent assay kits (Beyotime, Shanghai, China) according to the instructions.

### 4.9. Statistical Analysis

All results were analyzed using the *t*-test with GraphPad Prism 6 (GraphPad Prism version 6.00 for Windows, GraphPad Software, La Jolla California USA). Data are expressed as the mean ± SEM, and the statistical differences between groups are expressed as *p*-values, where *p* < 0.05 was considered statistically significant. The significance is expressed as * *p* < 0.05, ** *p* < 0.01, and *** *p* < 0.001; # *p* < 0.05, ## *p* < 0.01, and ### *p* < 0.001; and & *p* < 0.05, && *p* < 0.01, and &&& *p* < 0.001.

## 5. Conclusions

In summary, curcumin can inhibit intestinal pyroptosis through activation of the SIRT1/NRF2 pathway; reduce intestinal tissue secretion of IL-1β, IL-6, IL-18, and TNFα; and attenuate the loss of gastrointestinal barrier proteins ZO-1 and occludin, elucidating that curcumin is dependent on the gut–brain axis and thus exerted neuroprotective effects in the MPTP-induced PD mouse model (Graphical Abstract I). These results have important implications for revealing the pathogenesis of PD, and this study provides a potential opportunity to develop new preventive measures and therapeutic regimens that can target the gut–brain axis in the context of PD and provide a new intervention for the treatment of Parkinson’s disease.

## Data Availability

The data that support the findings of this study are available from the corresponding author, Tao Chen, upon reasonable request.
